# Recurrent desmoid tumors with ureteric fistula: a case report and literature review

**DOI:** 10.3389/fped.2025.1573732

**Published:** 2025-04-29

**Authors:** LongDi Yu, Chi Yuan, Kai Wang, JiaJie Li, Xue Ma

**Affiliations:** West China Hospital, Sichuan University, Chengdu, China

**Keywords:** desmoid tumors, adolescence, ureteric fistula, percutaneous nephrostomy, case report

## Abstract

**Background:**

Desmoid tumors (DT) is a rare soft tissue tumor classified as a borderline neoplasm. It exhibits locally aggressive growth and is prone to postoperative recurrence.

**Case presentation:**

We reported the first case of adolescent pelvic DT ureteral fistula, and report the patient's all progress of diagnosis and treatment. A recurrent pelvic DT in an adolescent female patient who initially underwent surgical resection but experienced recurrence, was effectively treated with sorafenib. Later, however, the patient's tumor changed from solid to cystic solid mixed, increasing in size, and the patient developed right hydronephrosis and hydroureter. Patients who underwent right sided antegrade pyelography were diagnosed with right sided ureteral desmoid tumor fistula and urinary fistula-related pseudocysts. Following treatment with right sided percutaneous nephrostomy, their condition improved, with resolution of right sided hydronephrosis and hydroureter, as well as partial shrinkage of the cystic component.

**Conclusion:**

DT can rarely result in the development of DT-ureteral fistulas. For patients with DT responsive to medical therapy, physicians should closely monitor tumor progression and pay attention to its invasion of adjacent organs and potential complications. Physicians need to balance therapeutic efficacy and quality of life when making treatment strategies for young patients.

## 1 Introduction

Desmoid tumors (DT), also known as aggressive fibromatosis (AF) or desmoid-type fibromatosis (DTF), is a rare soft tissue tumor originating from mesenchymal tissue and characterized by abnormal monoclonal fibroblast proliferation. The annual incidence is estimated at 2.4–4.3 cases per million, with 80% of patients being female, predominantly women of reproductive age (1, 2). Its development is closely associated with factors such as trauma, surgery, estrogen levels, and pregnancy. DT commonly occurs in the abdominal wall, intra-abdominal, or extra-abdominal regions. While they exhibit locally aggressive behavior, distant metastasis is rare. Some DTs have a self-limiting nature, and approximately 30%–40% of DTs will recur locally after surgery (1, 2). Clinical management strategies for DT include watchful waiting, surgical intervention, radiation therapy, and systemic treatments such as hormonal therapy, targeted therapy, and chemotherapy (2).

Intra-abdominal DTs can compress adjacent organs, leading to symptoms such as abdominal pain, loss of appetite, bloating, intestinal obstruction, and flank pain. Conditions such as hydronephrosis and hydroureter may also occur. DT-ureteral fistula is extremely rare, with only five cases reported in the literature to date (3–6), and no cases have been documented in adolescent patients with DT.

We report the first case of pelvic DT complicated with ureteral fistula in adolescence, the patient's all progress of diagnosis and treatment, and reviews five additional similar cases from the literature, aiming to provide insights for clinical diagnosis and treatment.

## 2 Case representation

**Figure 1:**
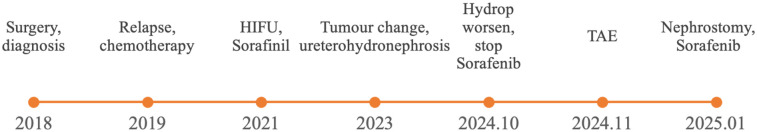
Timeline.

The patient is a 13-year-old Asian female, 160 cm in height and weighing 46 kg, admitted with a chief complaint of right side hydronephrosis and hydroureter persisting for over 2 years with a history of pelvic DT. Figure 1 shows the timeline of the diagnosis and treatment of this patient.

In 2018, the patient found a mass in the right inguinal area, which was shown to be deep into the obturator by CT (Supplementary Figure 1). Then, the right obturator tumor (10 cm × 8 cm × 6 cm) was completely surgically removed and diagnosed as a DT by pathological examination. Due to the family's inadequate understanding of the disease, they believed that complete resection equaled a cure. Furthermore, as the child was a left-behind child, no adjuvant therapy or follow-up was conducted after the surgery.

In 2019, a DT in the right pelvic area relapsed (Supplementary Figure 2), at a location different from that of the initial tumor. Then Vinorelbine combined with Methotrexate was used for 14 cycles, and the tumor continued to grow and the treatment failed.

In 2021, High-Intensity Focused Ultrasound (HIFU)-guided radiofrequency ablation treatment failed. Treatment with sorafenib was started, and the tumor size gradually reduced, while the right renal pelvis expanded.

In 2023, MRI showed a 6.3 cm × 6.1 cm × 3.8 cm cystic component appeared in the upper part of solid tumors, with internal spacing, accompanied by mild right hydronephrosis and hydroureter. And sorafenib treatment was continued.

In October 2024, CT showed a cystic mixed mass in the right pelvic area (10.5 cm × 5.2 cm), closely related to tissues such as the uterus, right appendix, bladder, right iliac blood vessels and pelvic muscles. And right hydronephrosis and hydroureter progressed. Therefore sorafenib treatment was stopped.

In November 2024, transcatheter arterial embolization (TAE) was performed, but the tumor showed no significant changes.

In January 2025, a right nephrostomy was performed, and the dose of sorafenib was increased. Finally, the cystic part of the tumor was reduced.

**Figure 2:**
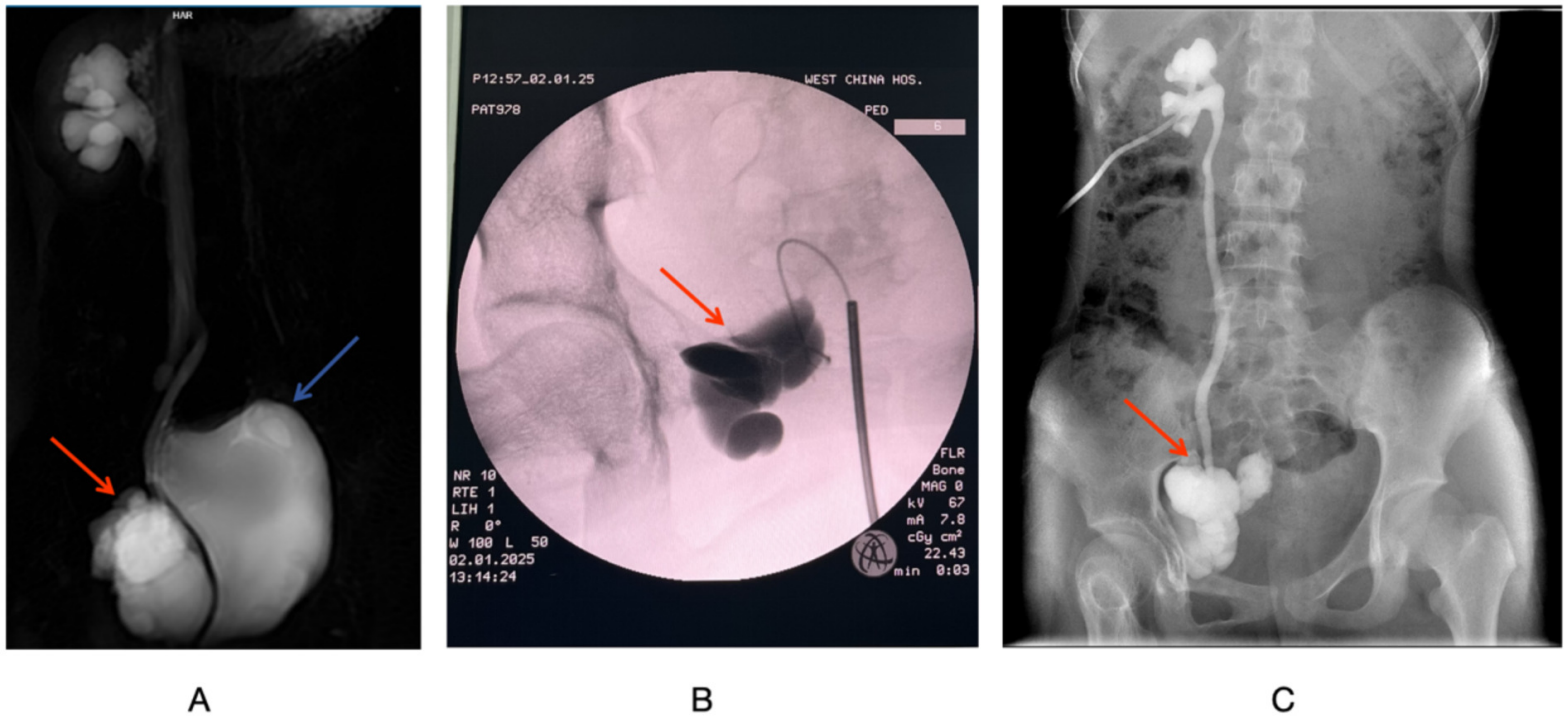
**(A)** MRI T2-weighted coronal imaging of the urinary system. **(B)** Intraoperative retrograde pyelography via the ureter. **(C)** Right side antegrade pyelography. The red arrow indicates the DT, and the blue arrow points to the bladder.

The patient's menstrual cycle is 28 days, each lasting 4–5 days, unmarried and childless, and there is no family history or genetic history of disease. The patient's abdomen is flat and soft without tenderness, rebound pain, or hepatosplenomegaly. But the lump is palpable in the lower right abdomen and has a soft texture. Blood counts, biochemistry, tumor markers and estrogen levels are normal. SPECT renal scintigraphy using 99mTc-DTPA showed normal GFR in both kidneys and poor drainage in the right upper urinary tract. MRI of the abdomen with contrast showed an irregular cystic mass (10.5 cm × 5.6 cm) in the right pelvis with an internal solid component (Figure 2A). The cyst wall, diaphragm, and solid portion show increased heterogeneity, and the lesion appears to communicate distal to the right ureter (Supplementary Figure 3), causing mild hydronephrosis and right upper hydroureters. The mass is indistinguishable from the middle and distal vessels of the right ilium, and the lumen of the vessel is not clear. Pathological examination showed morphology consistent with DT, immunohistochemistry showed β-catenin (partially nuclear positive), and genetic testing revealed a CTNNB1 T41A mutation. The final diagnosis confirmed DT.

A multidisciplinary team (MDT) consisting of pediatric surgery, oncology, gynecology, pathology, and radiology recommends an attempt to place a right ureteral stent to relieve right urinary tract obstruction along with systemic treatment of right pelvic DT.

Under general anesthesia, the patient underwent right sided pyelogram, ureteroscopy, and percutaneous nephrostomy. During the procedure, the ureteroscope is advanced approximately 3.5 cm to the right ureter, but is blocked by tortuosities and folds. A 0.035 cm hydrophilic guidewire is inserted, but the renal pelvis cannot be reached. A 14F ureteral catheter was placed, and contrast imaging showed that Iohexol had accumulated in the pelvic mass without entering the ureters or renal pelvis (Figure 2B). Throughout the procedure, the right ureter remained intact without any injury. Percutaneous nephrostomy was completed on the right kidney under ultrasound guidance, draining approximately 50 ml of pale yellow urine.

Postoperatively, antibiotics are given to prevent infection and fluids and electrolytes are replaced. The amount of urine drained from the right nephrostomy tube per day is approximately equal to the amount of urine self-free, and the total daily urine output is approximately 2,000 ml. The urine is pale yellow and transparent, with no flocculent substance. On postoperative day 5, anterograde pyelogram showed visualization of the right kidney, ureter, and tumor area, with no contrast leakage in the abdominal or pelvic cavities, confirming the presence of the DT fistula and the integrity of the right ureter. Evidence of hydronephrosis in the right kidney and ureters (Figure 2C). On postoperative day 7, the patient continue oral sorafenib treatment with an adjusted dose of 400 mg once daily according to the her body surface area. Apart from this, the patient received no other treatments.

One month postoperatively, MRI (Supplementary Figure 5): the volume of cystic components was smaller than that before the right nephrostomy, and the hydrops in the right kidney and ureters were relieved. The solid volume of the tumor appears to be smaller than before, and follow-up is still required.

In the future, the patient will continue to monitor the abdominal pelvic CT. After the cystic components and tumor tissue are further reduced, the patient will be evaluated for the right vesicureter re-implantation surgery.

## 3 Discussion and conclusion

We report the first case of DT ureteral fistula in an adolescent patient and comprehensively document disease progression and prognosis, filling the gap of previously unclear prognosis. This patient has a complex medical history, and after various treatment regimens, the disease progression is well controlled, which provides a reference for the diagnosis and treatment of such patients.

DT ureteral fistulas are extremely rare, and Table 1 summarizes the reporting cases. Imaging examinations, such as IVU, CT, and SPECT renal scans, have confirmed urine infiltration into the tumor tissue, resulting in cystic-solid changes and accompanying by hydronephrosis and hydroureter (3–6). All patients underwent either double-J stent placement or nephrostomy, but prognoses varied widely.

**Table 1:** Literature review of abdominal desmoid tumor-ureteral fistula.

Publication year	Authors	Age/sex	Presentations	Diagnosis	Techniques	Treatments	Follow-up
1997	Richard et al.	34/F	R-upper abdominal mass	ADT, R-UD	IVU	R-PCN, R-DJS	Routine follow-up
1997	Richard et al.	45/M	L-ureteral obstruction	GS, ADT, L-PH, L-DT-UF	IVU	R-PCN, L-DJS	/
2006	Lath et al.	17/F	Fullness, colicky abdominal pain, fatigue, weight loss	MF, R-HUH, R-DT-UF	CT	R-DJS, no systemic therapy	Death
2016	Yoo et al.	35/F	Abdominal pain	GS, MF, R-HD, B-DT-UF	SPECT	R-PCN, Pharmacotherapy	Routine follow-up
2016	Kim et al.	20/F	Abdominal pain	GS, ABD, L-UD, L-DT-UF	SPECT	L-PCN, L-RA	/

R, right side; L, left side; B, bilateral sides; ADT, abdominal desmoid tumor; UD, ureteral dilation; GS, Gardner's syndrome; PH, pelvic hydronephrosis; DT-UF, desmoid tumor-ureteral fistula; MF, mesenteric fibromatosis; HUH, hydroureteronephrosis; HD, hydronephrosis; IVU, intravenous urography; CT, computed tomography; SPECT, single photon emission computed tomography; PCN, percutaneous nephrostomy; DJS, double-J stent; RA, renal autotransplantation.

Studies have shown that the overall recurrence rate of DF after treatment is approximately 30%–40% (7), with a recurrence rate of around 30%–50% after surgery (7, 8). Incomplete excision (R2 resection) is associated with a higher risk of recurrence and progression compared to complete excision (R0, R1) (9–11). For recurrent patients, positive surgical margins often indicate a poor prognosis (12). For progressing or recurrent pelvic desmoid fibromatosis, drug therapy is the preferred treatment (13). Moreover, surgery and trauma can promote tumor growth, leading to recurrence (14–16). And mutations in β-catenin have also been associated with recurrence (13, 17–19). In children, DT are more aggressive (20), and the recurrence rate is relatively higher (21), therefore surgery should be avoided possibly.

In this case, prior to the formation of the DT-ureteral fistula, sorafenib treatment had been proven effective for the patient. And sorafenib has been shown to be highly effective in recurrent DT, and has gradually become a first-line treatment option for DTs (22, 23). During sorafenib treatment, the patient developed a DT-ureteral fistula. sorafenib primarily exerts its effects by inhibiting vascular endothelial growth factor receptor (VEGFR) and other pathways (24), which may suppress the angiogenesis of normal tissues, such as the ureter, leading to ureteral injury. However, there have been no reported adverse effects related to sorafenib-induced ureteral tissue damage, and further research is needed to explore this potential association.

For young patients with ureteral obstruction caused by desmoid tumor ureteral fistula, double J tubes or percutaneous nephrostomy should be actively placed to relieve the obstruction, and systemic therapy should be actively used to create conditions for final surgical reconstruction of the urinary tract.

## Data Availability

The original contributions presented in the study are included in the article/Supplementary Material, further inquiries can be directed to the corresponding author.

## Supplementary material

Supplemnetary Figure 1 MRI on 2018-01-31: An irregular mass with abnormal signal intensity was identified within the soft tissue of the right pelvic floor and medial upper thigh. Malignancy is highly suspected. The lesion involves the right obturator region, obturator muscle, and adjacent pubic and ischial bones, with abnormal signal intensity noted in these areas. The red arrow indicates a desmoid tumor.

Supplementary Figure 2 MRI on 2021-01-08: The desmoid tumor is located within the pelvic cavity and compresses the pelvic floor muscle group. The red arrow indicates the desmoid tumor.

Supplementary Figure 3 MRI on 2024-12-31: Plain and enhanced scans of the lower abdomen and pelvis revealed a high possibility of ureteral involvement by the tumor (with possible urine leakage?). The red arrow indicates the desmoid tumor, and the blue arrow indicates the right ureter.

Supplementary Figure 4 MRI on 2024-12-31: Axial imaging: The red arrow indicates the desmoid tumor, and the blue arrow indicates the bladder.

Supplementary Figure 5 MRI on 2025-02-26: One-month postoperative follow-up revealed a mass lesion in the right pelvic wall and medial thigh muscle interstitial area. Compared with the previous imaging, the tumor volume has decreased. The red arrow indicates the desmoid tumor.
