# Supplementary figures and images for: Recurrent desmoid tumors with ureteric fistula: a case report and literature review
Source: Front Pediatr. 2025 Apr 29;13:1573732. doi: 10.3389/fped.2025.1573732 (PMC12069339; doi:10.3389/fped.2025.1573732)

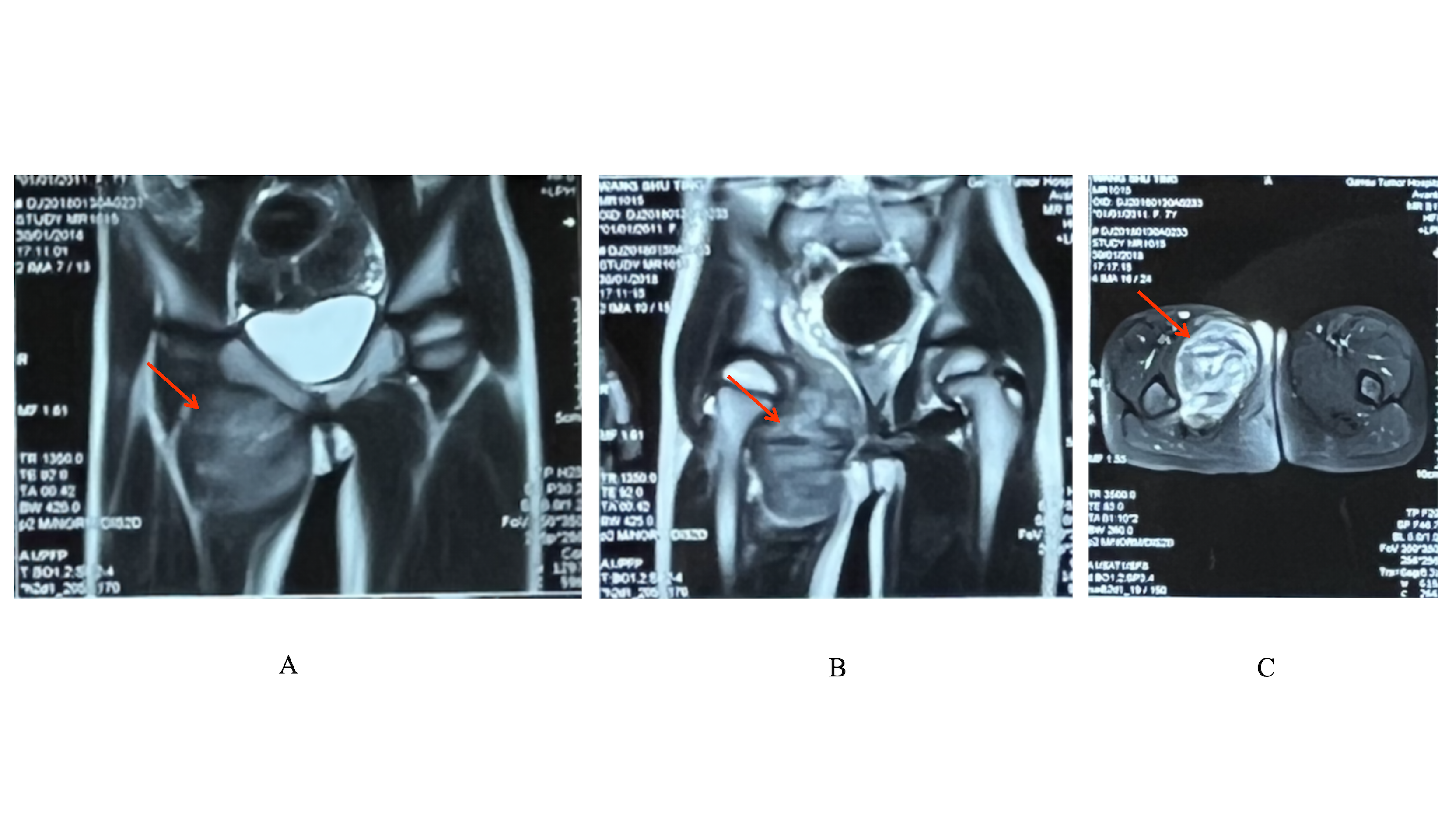

Supplement: Supplemnetary Figure 1 — MRI on 2018-01-31: An irregular mass with abnormal signal intensity was identified within the soft tissue of the right pelvic floor and medial upper thigh. Malignancy is highly suspected. The lesion involves the right obturator region, obturator muscle, and adjacent pubic and ischial bones, with abnormal signal intensity noted in these areas. The red arrow indicates a desmoid tumor. [file Image1.tif]

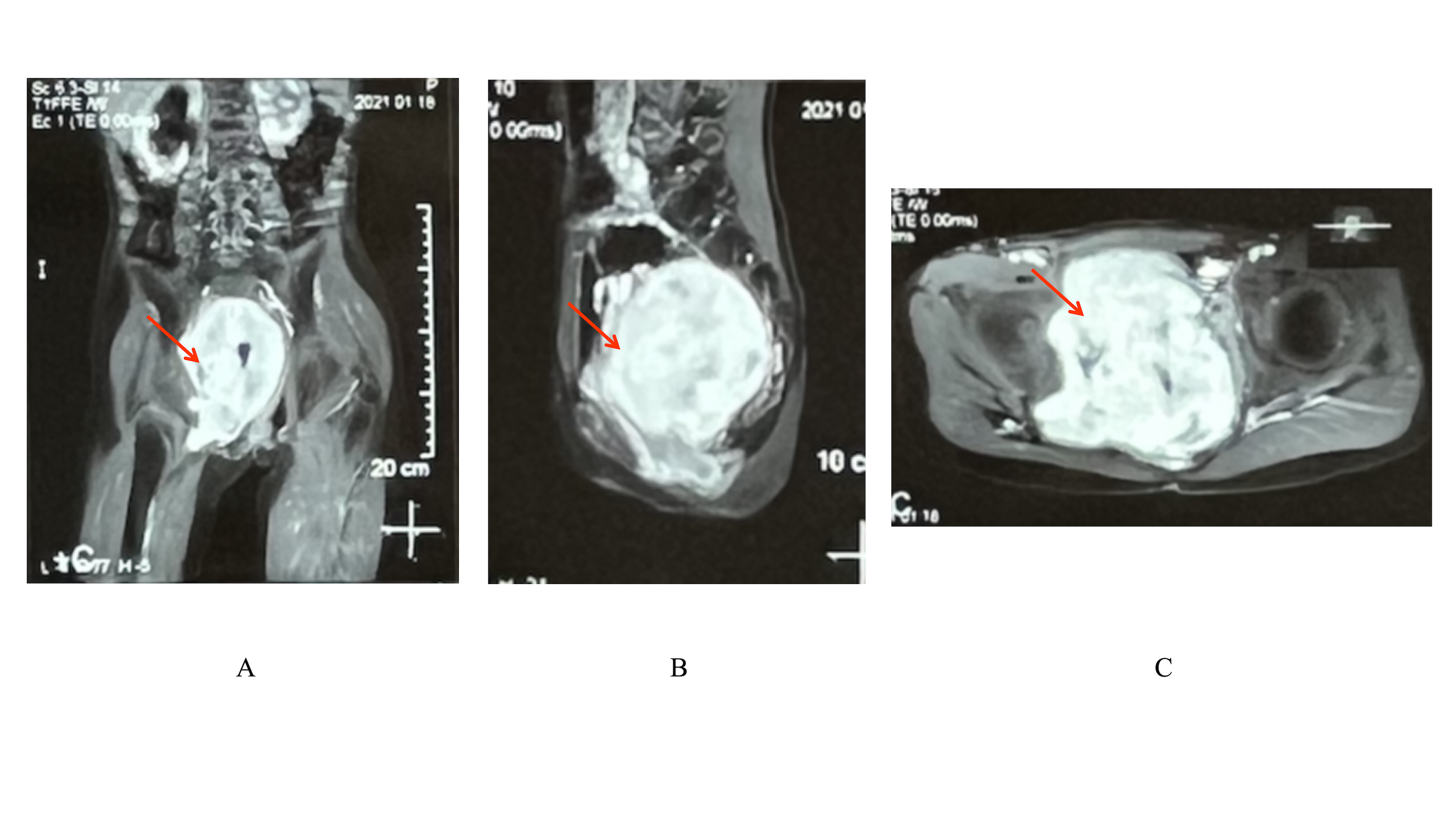

Supplement: Supplementary Figure 2 — MRI on 2021-01-08: The desmoid tumor is located within the pelvic cavity and compresses the pelvic floor muscle group. The red arrow indicates the desmoid tumor. [file Image2.tif]

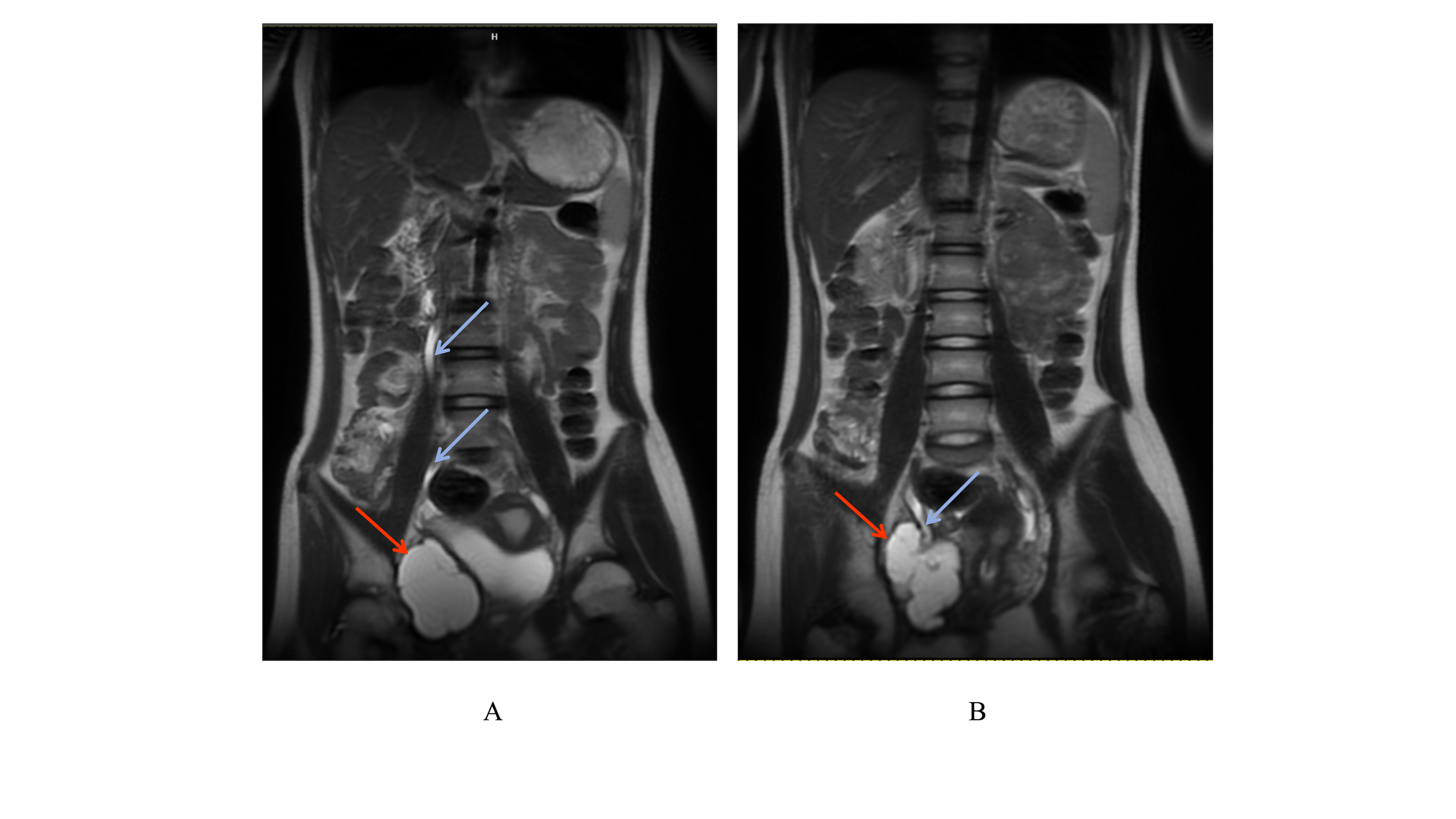

Supplement: Supplementary Figure 3 — MRI on 2024-12-31: Plain and enhanced scans of the lower abdomen and pelvis revealed a high possibility of ureteral involvement by the tumor (with possible urine leakage?). The red arrow indicates the desmoid tumor, and the blue arrow indicates the right ureter. [file Image3.tif]

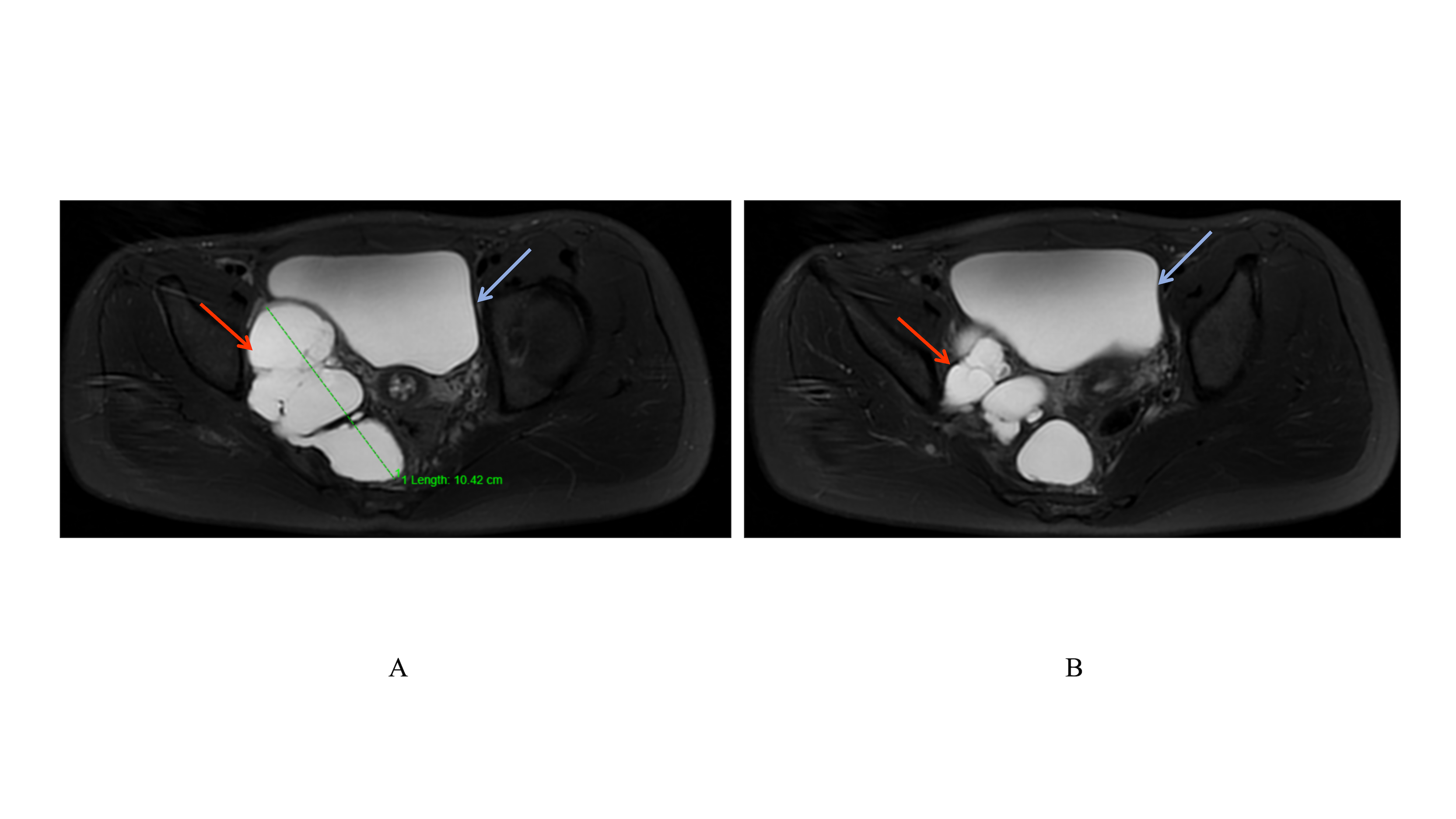

Supplement: Supplementary Figure 4 — MRI on 2024-12-31: Axial imaging: The red arrow indicates the desmoid tumor, and the blue arrow indicates the bladder. [file Image4.tif]

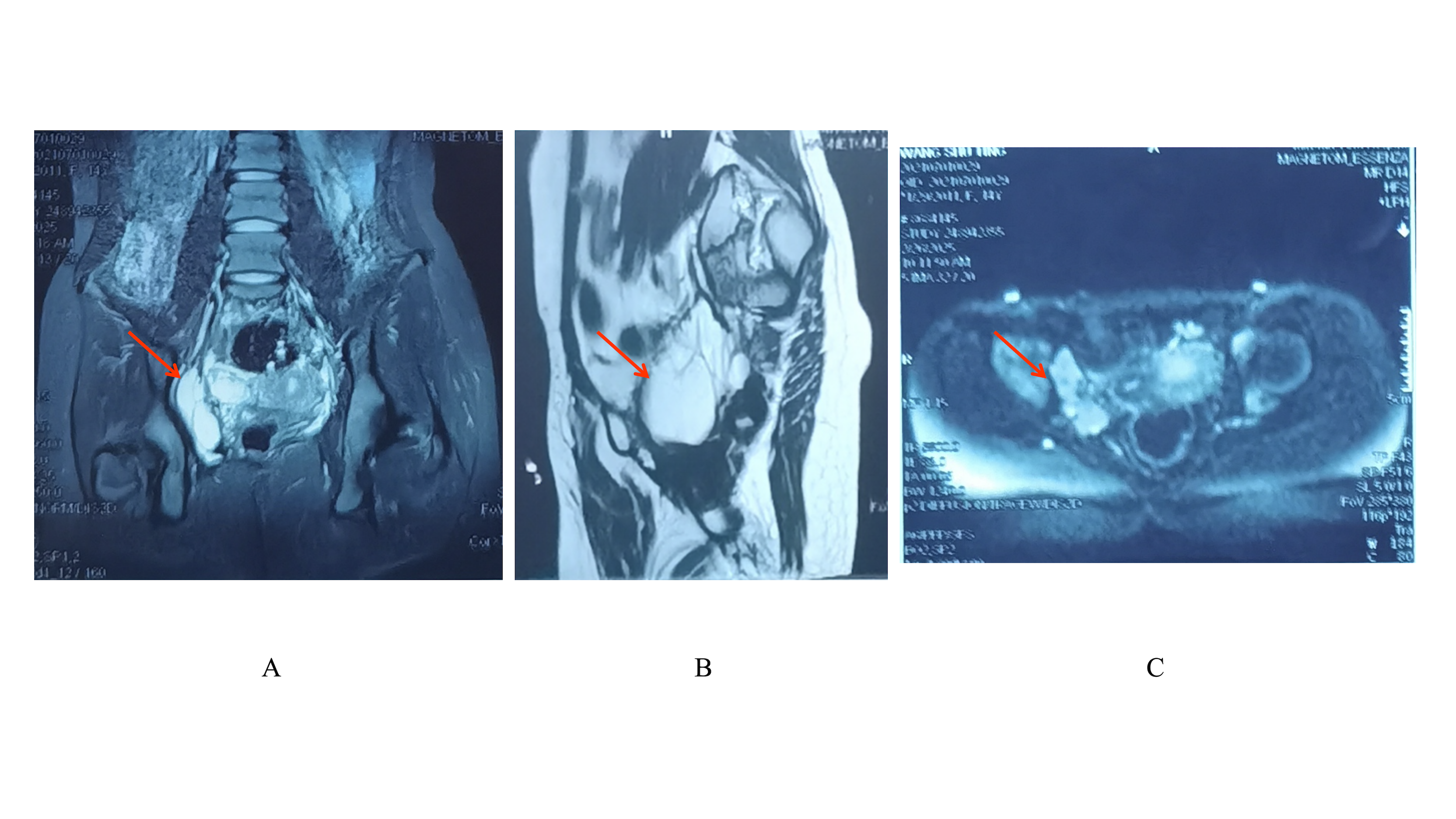

Supplement: Supplementary Figure 5 — MRI on 2025-02-26: One-month postoperative follow-up revealed a mass lesion in the right pelvic wall and medial thigh muscle interstitial area. Compared with the previous imaging, the tumor volume has decreased. The red arrow indicates the desmoid tumor. [file Image5.tif]
